# The COLON study: *Co*lorectal cancer: *L*ongitudinal, *O*bservational study on *N*utritional and lifestyle factors that may influence colorectal tumour recurrence, survival and quality of life

**DOI:** 10.1186/1471-2407-14-374

**Published:** 2014-05-27

**Authors:** Renate M Winkels, Renate C Heine-Bröring, Moniek van Zutphen, Suzanne van Harten-Gerritsen, Dieuwertje EG Kok, Fränzel JB van Duijnhoven, Ellen Kampman

**Affiliations:** 1 Division of Human Nutrition, Wageningen University, Wageningen, The Netherlands; 2 Department for Health Evidence, Radboud UMC Nijmegen, Nijmegen, The Netherlands

**Keywords:** Colon cancer, Rectal cancer, Nutrition, Diet, Dietary supplements, Survival, Recurrence, Cohort, Body composition, Quality of life (max 10)

## Abstract

**Background:**

There is clear evidence that nutrition and lifestyle can modify colorectal cancer risk. However, it is not clear if those factors can affect colorectal cancer treatment, recurrence, survival and quality of life. This paper describes the background and design of the “COlorectal cancer: Longitudinal, Observational study on Nutritional and lifestyle factors that may influence colorectal tumour recurrence, survival and quality of life” – COLON – study. The main aim of this study is to assess associations of diet and other lifestyle factors, with colorectal cancer recurrence, survival and quality of life. We extensively investigate diet and lifestyle of colorectal cancer patients at diagnosis and during the following years; this design paper focusses on the initial exposures of interest: diet and dietary supplement use, body composition, nutrient status (e.g. vitamin D), and composition of the gut microbiota.

**Methods/Design:**

The COLON study is a multi-centre prospective cohort study among at least 1,000 incident colorectal cancer patients recruited from 11 hospitals in the Netherlands. Patients with colorectal cancer are invited upon diagnosis. Upon recruitment, after 6 months, 2 years and 5 years, patients fill out food-frequency questionnaires; questionnaires about dietary supplement use, physical activity, weight, height, and quality of life; and donate blood samples. Diagnostic CT-scans are collected to assess cross-sectional areas of skeletal muscle, subcutaneous fat, visceral fat and intermuscular fat, and to assess muscle attenuation. Blood samples are biobanked to facilitate future analyse of biomarkers, nutrients, DNA etc. Analysis of serum 25-hydroxy vitamin D levels, and analysis of metabolomic profiles are scheduled. A subgroup of patients with colon cancer is asked to provide faecal samples before and at several time points after colon resection to study changes in gut microbiota during treatment. For all patients, information on vital status is retrieved by linkage with national registries. Information on clinical characteristics is gathered from linkage with the Netherlands Cancer Registry and with hospital databases. Hazards ratios will be calculated for dietary and lifestyle factors at diagnosis in relation to recurrence and survival. Repeated measures analyses will be performed to assess changes over time in dietary and other factors in relation to recurrence and survival.

## Background

Colorectal cancer is the third most common type of cancer worldwide [1]. Lifestyle and nutritional factors influence colorectal cancer risk. High consumption of red and processed meat and alcoholic beverages and low consumption of foods containing dietary fibre convincingly increase the risk of colorectal cancer. Body fatness – especially abdominal fatness-, and adult attained height increase the risk of colorectal cancer, while physical activity protects against colorectal cancer [2,3].

In contrast to the extensive knowledge on the role of nutrition and lifestyle in the prevention of colorectal cancer, much less is known about the role of diet and lifestyle during and after treatment of colorectal cancer. Few prospective studies reported on factors that were associated with colorectal cancer recurrence and survival, while those studies were often hampered by the fact that dietary assessment was retrospective, that patient groups were small and heterogeneous, or that other prognostic factors were not taken into account [4]. Evidence-based lifestyle recommendations are necessary for the increasing number of colorectal cancer survivors, since these survivors may show a major interest in adjusting their usual habits [5-7]. The aim of the current study is to further explore the association between diet and other lifestyle factors in colorectal cancer prognosis, survival and quality of life, with special emphasis for the role of diet and dietary supplement use, body composition, nutrient status, and composition of the gut microbiota.

Few prospective studies assessed the association between diet and dietary supplement use and colorectal cancer prognosis and survival. An observational study within a randomized controlled chemotherapy trial (n = 1,009 stage III colorectal cancer patients) [8], showed that colorectal cancer patients who scored high on a diet that could be described as a Western diet, with high intakes of meat, fat, refined grains, and desserts, had a 3 times higher risk of cancer recurrence or death (HR 3.25 (2.04-5.19)) than persons who scored low on such a pattern. Conversely, a prudent pattern, high in vegetables, fruits, poultry, and fish, was not associated with colorectal cancer outcomes in that study. There are only few additional publications on diet and colorectal cancer outcomes [4]. It is unclear if the use of dietary supplements by colorectal cancer patients affects colorectal cancer recurrence and survival. Dietary supplement use among patients has been assessed in several –mainly US – studies and is estimated to be as high as 60-80% [9]. An observational study, again within a randomized controlled chemotherapy trial (n = 1,038 stage III patients) showed that multivitamin use during and after adjuvant chemotherapy was not significantly associated with outcomes in patients with stage III colon cancer [10]. It has been hypothesized that folic acid supplementation, may be involved in progression of established neoplasms [11]. This stresses the need to further address the role of dietary supplement use during and after colorectal cancer treatment.

Some data suggest that colorectal cancer patients who are obese or underweight may experience higher mortality rates than normal and overweight patients [4,12-15], however study results are not consistent. Underweight, overweight and obesity are usually only assessed by measuring the body mass index (BMI) [16-18], while BMI is not a valid measure for fat distribution or body composition [19]. Muscle depletion – assessed from diagnostic computed tomography (CT)-scans - has been associated with worse survival in a mixed groups of cancer patients (n = 1,400), independently of BMI [20]. Moreover, among obese patients, those who are sarcopenic – i.e. those with severe muscle depletion- appear to have worse survival than patients who are not-sarcopenic [21]. This warrants further study on the association between muscle mass, fat mass and survival among cancer patients. In addition, fat distribution of abdominal fat is an area that requires further investigation. Abdominal fat is mainly divided into two depots: subcutaneous and intra-abdominal or visceral fat. Visceral fat accumulation has been associated with increased incidence of colorectal cancer [5]; its association with recurrence of colorectal cancer has only sparsely been studied in small studies with short follow-up [22-25]. Nevertheless, those studies suggest that increased visceral fat areas, or an increased visceral fat vs subcutaneous fat ratio may increase the risk of recurrence. Visceral adiposity may also unfavourably affect colorectal cancer survival, but again this has only been studied in small populations (50–200 patients) with short follow-up and mostly in patients with metastatic disease [22-24,26]; results were therefore not conclusive. Concluding, the associations of body composition and fat distribution with recurrence and survival of colorectal cancer patients are promising areas of investigation.

Nutrient status at diagnosis as well as during treatment may also affect recurrence and survival. For instance, the role of vitamin D in colorectal cancer prevention and survival has gained much interest in recent years. A recent meta-analysis suggested that higher 25(OH)D levels (>75 nmol/L) were associated with significantly reduced mortality in patients with colorectal cancer [27]. Results should be interpreted with caution, as the assessment of 25(OH)D levels differed between the individual studies of the meta-analysis (pre vs post-diagnostic). Moreover, most studies only have one measurement of vitamin D levels, while cancer treatment and stage of disease may have a large impact on vitamin D status. Thus, cohort studies with repeated measurement of vitamin D levels are urgently needed.

Many colorectal cancer patients treated with chemotherapy suffer from mucositis and gastrointestinal complaints, such as severe diarrhoea, nausea and vomiting [28,29]. Knowledge on the role of the gut microbiota - a major compartment of the gastrointestinal tract- in human health has emerged in the past years [30]. Yet, the gut microbiota has been relatively ignored in studies focusing on the pathophysiology and side-effects of cancer therapies [31]. There is some evidence that chemotherapy induces a large decline in the diversity of the gut microbiota [32,33]. To what extent colorectal cancer patients receiving chemotherapy experience similar declines in diversity and whether diet and lifestyle affect recovery of the gut microbiota during and after chemotherapy is largely unknown.

In this study, we will assess associations of diet and other lifestyle factors, with colorectal cancer recurrence and survival and with quality of life. We comprehensively investigate diet and lifestyle of colorectal cancer patients at diagnosis and during the following years. This design paper focusses on the four initial topics of interest in this prospective cohort study: diet and dietary supplement use, body composition, nutrient status (e.g. vitamin D), and composition of the gut microbiota.

## Methods/design

The “Colorectal cancer: Longitudinal, Observational study on Nutritional and lifestyle factors that influence colorectal tumour recurrence, survival and quality of life” – COLON - study is a prospective observational cohort study that aims to include at least 1,000 colorectal cancer patients from regional and academic hospitals in the Netherlands over a period of ~5 years. Ethical approval for the study was granted by the Committee on Research involving Human Subjects, region Arnhem-Nijmegen (Commissie Mensgebonden Onderzoek – CMO, region Arnhem Nijmegen).

### Recruitment

Men and women of all ages, who were newly diagnosed with colorectal cancer (ICD codes C18-20) in any stage of the disease in one of the 11 participating hospitals, are eligible for the study. Non-Dutch speaking patients, or patients with a history of colorectal cancer or (partial) bowel resection, chronic inflammatory bowel disease, hereditary colorectal cancer syndromes (Lynch syndrome, FAP, Peutz-Jegher), dementia or another mental condition that makes it impossible to fill out questionnaires correctly, will be excluded from the study. Recruitment is conducted in close cooperation with staff of the oncology, gastroenterology and/or internal medicine departments of the participating hospitals. Recruitment procedures vary slightly per hospital. In general, eligible patients receive an information leaflet about the COLON study from their treating physician or from the nurse-practitioner shortly after diagnosis during a routine clinical visit. Patients can consult with their physician or nurse-practitioner, with a member of the study team, and/or with an independent physician if they have questions about the study. Patients who agree to participate have to provide written informed consent.

### Data collection

**Figure 1:**
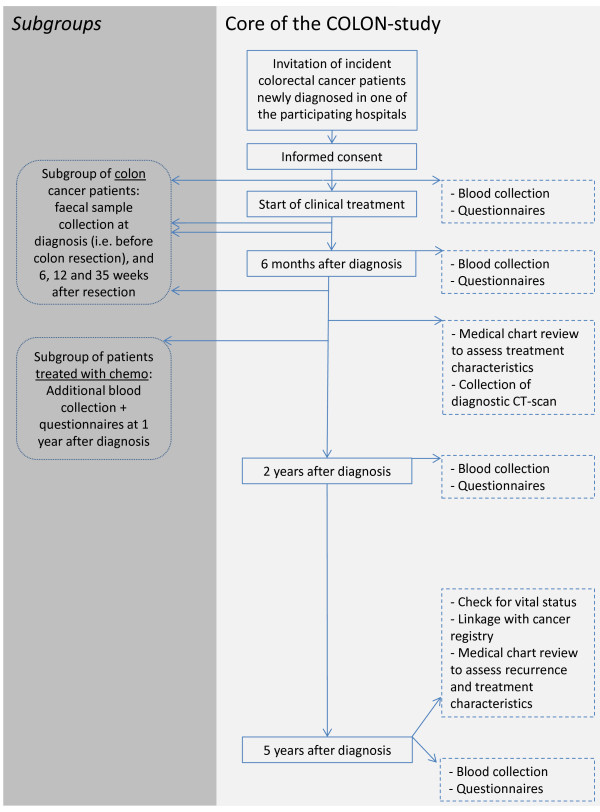
Overview and design of the COLON study.

Patients are asked to fill out several questionnaires upon recruitment (at diagnosis), at 6 months, 2 years and 5 years after recruitment (Figure 1). In addition, participants are asked to donate a blood sample at each time point. Patients who are treated with chemotherapy, are asked to additionally fill out questionnaires and to donate an extra blood sample 1 year after recruitment. At that point in time most of those patients will have completed their treatment, while other patients will have finished their treatment within 6 months. Patients are asked for permission for collection of paraffin-embedded tumor-material using the nationwide network and registry of histo- and cytopathology in the Netherlands (PALGA).

### Demographic and health characteristics

Demographic and health characteristics are assessed with a self-administered lifestyle questionnaire containing questions on demographics (education, ethnicity, living situation, number of children), body weight and height, history of body weight, smoking habits, history of medication (including use of aspirin and other NSAIDs), family history of cancer, any changes that patients made to their diet because of bowel complaints or other reasons, type of (alternative) treatment, experienced side-effects of treatment, comorbidities, and for women: menopausal status, menstrual and reproductive history.

### Dietary intake & dietary supplement use

Habitual dietary intake in the month preceding diagnosis - and for the other time-points the preceding month -, is assessed using a semi-quantitative food frequency questionnaire. This questionnaire was previously validated [34,35], and slightly adapted to be able to distinguish meat intake with respect to red, processed, and white meat, and for dairy to be able to distinguish fermented and unfermented dairy. For all items, frequencies per day and standard portion sizes will multiplied to obtain intake in grams per day. Energy intake and nutrient intakes will be calculated using the Dutch food composition table [36]. Additionally, the food frequency questionnaire contains questions on the use of organic foods, i.e. the type of organic foods and the frequency of use.

Dietary supplement is assessed using a self-administered dietary supplement questionnaire developed by the Division of Human Nutrition of Wageningen University, the Netherlands. The dietary supplement questionnaire contains questions on use of multivitamin/minerals supplements and other mixtures not classified as multivitamins/minerals (e.g. vitamin B-complex, antioxidant mixtures, combination of vitamin A/D, mixture of calcium/magnesium/zinc, other mixtures), and supplemental vitamin A, folic acid, vitamin B12, vitamin C, vitamin D, vitamin E, calcium, magnesium, zinc, iron, selenium, chrome, fish oil, and herbal and specialty supplements, and on the dosage and frequency of intake. Upon recruitment, participants are asked whether they used any dietary supplement during the year before colorectal cancer diagnosis. At the other time-points, dietary supplement use in the period since the last questionnaire is enquired.

### Body composition

Patients are asked to measure and report their waist and hip circumference; instructions and a measuring device are provided. In addition, CT-images are retrieved from medical records of all participants for the assessment of body composition. Diagnostic CT-images are available from almost all colorectal cancer patients (~85-90%), as they are used for diagnosis and staging of the disease. From these CT-images, cross-sectional areas (cm^2^) of skeletal muscle, subcutaneous fat, visceral fat and inter-muscular fat will be quantified at the landmark level of the third lumbar vertebra (L3) using Slice-O-matic software (Tomovision, Canada). Cross-sectional L3 adipose and muscle areas are linearly related to total body adipose and muscle mass [37-39].

### Physical activity

Self-reported physical activity is assessed using the Short Questionnaire to ASsess Health-enhancing physical activity (SQUASH) [40]. The general purpose of this questionnaire is to assess habitual physical activity, with a reference period of a normal week in the past months. Participants are asked to report their average time spend on the following pre-structured types of activities: commuting activities, activity at work, household activities and leisure time activities (walking, bicycling, gardening, odd jobs and up to four sports). The SQUASH consists of three main queries: days per week, average time per day, and intensity. The recorded activity will be converted into Metabolic Equivalent (MET)-scores using the Compendium of Physical Activities [41]. Validation studies [40,42,43] showed that the SQUASH-questionnaire is fairly reliable and reasonably valid in an adult population and may be used to rank participants based on their physical activity level and to categorize them according to the Dutch physical activity guideline (30 minutes or more of at least moderate intense physical activity for a minimum of 5 days per week).

### Blood sample collection and analysis

Non-fasting blood samples are drawn from patients upon recruitment and at all later time-points during a regular clinical visit of the patient. The baseline blood sample is preferably taken before surgery or start of treatment. In case of neo-adjuvant radiation therapy, it is not always possible to draw blood before the start of treatment, and for those patients a blood sample is collected before surgery. For each blood sample, haematocrit is assessed immediately after blood draw at all study sites. Blood samples are processed into serum (6 aliquots), plasma (5 aliquots), full blood (2 aliquots), and buffy coat (2 aliquots) and stored in a biobank at -80°C. All procedures are defined in a protocol in order to ensure standardisation over study sites. Blood samples are biobanked for later analysis of metabolites, biomarkers, nutrients etc. Analysis of 25-hydroxy vitamin D is already anticipated; in addition, metabolomics will be performed. Both 25-hydroxy vitamin D2 and 25-hydroxy vitamin D3 levels will be assessed in serum samples using a liquid chromatography tandem mass spectrometry method [44]. In a subset of the patients targeted and untargeted metabolomic analysis will be performed using the Biocrates AbsoluteIDQ p180 Kit for the targeted approach and UPLC-ESI-qTOF for the untargeted approach at the IARC, France.

### Faecal sample collection and analysis

In order to assess whether cancer therapy affects composition and function of the gut microbiota in colon cancer patients, faecal samples are collected from a subgroup of patients with colon cancer who are diagnosed in one of the participating hospitals (Hospital Gelderse Vallei, Ede). Faecal samples are collected shortly after diagnosis (i.e. before colon resection), and 6, 12 and 35 weeks after resection. For patients who are treated with chemotherapy, this corresponds to sample collection before, during and after chemotherapy. A phylogenetic microarray (the Human Intestinal Tract Chip; HITCHip) will be used for a high-throughput characterisation of the composition of the gut microbiota [45].

### Clinical outcome measurements

Information on clinical factors are retrieved from linkage with the Netherlands Cancer Registry and will include: pathologic and clinical disease stage (TNM), date of colorectal cancer incidence, location of the tumour, morphology, degree of differentiation, number of lymph nodes surgically sampled and number of positive lymph nodes, type and date of surgery, surgical complications (anastomotic leakage, abscess), tumour residue, type of treatment (chemotherapy, radiotherapy, chemoradiation, other) and date of start treatment, location of metastases (ICD-code) and distance of tumour from anus (rectal tumours only). Additional clinical data will be retrieved from medical record abstraction. We are using standardized forms and methods to abstract the medical records for all of the participants at regular intervals during the cohort study. Medical variables include history of gastro-intestinal disease, date and indication for endoscopy at diagnosis, length of hospital stay after primary surgery, body weight and height, size of the tumour, length of surgically removed bowel, CEA level, all treatment and follow-up care including data on chemotherapy and radiation therapy, adenoma/carcinoma recurrence.

The main outcomes of this cohort are treatment completion rates, side-effects of treatment, disease outcomes and quality of life. Disease outcomes are: colorectal cancer recurrence, colorectal adenoma occurrence/recurrence and survival/mortality. Information on mortality/survival is gathered from linkage with the Municipal Personal Records Database (in Dutch: Basisregistratie personen), information on cause of death is ascertained by linkage with Statistics Netherlands.

### Assessment of quality of life

Quality of life is assessed with the European Organization for Research and Treatment of Cancer Quality of Life Questionnaire C30 version 3.0 (EORTC QLQ-C30), which is a widely used measure of Health-Related Quality of Life in cancer [46,47]. The questionnaire contains five functioning scales (physical, role, cognitive, emotional, and social functioning); three symptom scales (fatigue, pain, and nausea and vomiting); and a global health and health related quality of life scale. Patient-reported chemotherapy-induced peripheral neuropathy is assessed in patients treated with chemotherapy using the “Quality of Life Questionnaire-CIPN twenty-item scale” (QLQ-CIPN20); this questionnaire is provided at the 1 year time-point [48,49]. This 20 item questionnaire includes three scales assessing sensory, motor and autonomic symptoms that can result from neuropathy.

An individual’s coping style is assessed with the “Coping Inventory for Stressful Situations”-CISS questionnaire [50], a valid and reliable tool to assess basic coping styles. This inventory measures three different coping styles: task-oriented, emotion-oriented and avoidance-oriented coping. Coping style is only assessed at the 2 year time-point, as this is considered to be a stable factor that will not change over time.

### Power considerations and data analysis

A prospective cohort study assesses multiple exposures and outcomes. The power calculation for this cohort study was based on one exposure that was of special interest in this study – dietary supplement use - and the anticipated association with recurrence of colorectal cancer and survival. There are few publications on the prevalence of dietary supplement use in the general population in the Netherlands, or among colorectal cancer patients; therefore, we assume that supplement use in patients is comparable to supplement use in the general elderly population: ~45% [51].

Our aim is to include at least 1,000 patients in our study. After 5 years of follow-up, we expect a number of 320 recurrences and 250 deaths [8,52]. This will enable us to detect the following associations: for recurrences, a HR of ≤0.78 or ≥1.31 (alpha = 0.05 and power = 0.8), for mortality, a HR of ≤0.77 or ≥1.33 (alpha = 0.05 and power = 0.8).

Cox proportional hazard models will be used to calculate hazard ratios for dietary and lifestyle factors at diagnosis in relation to outcomes. Changes of dietary and lifestyle factors over time will be analyzed with analysis techniques for longitudinal data, since the observations of one individual over time are not independent.

All associations will be adjusted for age and sex and if applicable for stage of the disease. Additionally, we will check whether other additional variables should be included in the multivariate models as potential confounding variables and/or effect measure modifiers.

## Discussion

This is the largest prospective European study among colorectal cancer patients with repeated information on a variety of lifestyle factors and other exposures. Recruitment is expected to be complete by the beginning of 2015. This prospective cohort study will shed further light on the associations between diet, other lifestyle factors and quality of life, recurrence and survival among colorectal cancer patients.

Although this is the largest European prospective study so far, even larger studies are necessary for specific analyses in subgroups of patients, e.g. within stages of disease, or within groups of patients with the same treatment. Therefore, we have harmonised our study protocol with two other ongoing prospective studies among colorectal cancer patients: the EnCoRe study of Maastricht University, the Netherlands [53] and with the ColoCare Study of the German Cancer Research Center in Heidelberg [54]. Thus, in future collaborations, we can pool the results of these studies to be able to increase the power; the expected number of patients in all three cohorts will be at least 2,200.

## Competing interests

The authors declare that they have no competing interests.

## Authors’ contributions

All authors contributed to the conception and design of the study. RW drafted the manuscript, all authors critically read and revised the manuscript. All authors approved the final version of the manuscript.

## Pre-publication history

The pre-publication history for this paper can be accessed here:

http://www.biomedcentral.com/1471-2407/14/374/prepub
